# Net CO_2_ assimilation rate response of tomato seedlings (*Solanum lycopersicum* L.) to the interaction between light intensity, spectrum and ambient CO_2_ concentration

**DOI:** 10.3389/fpls.2023.1327385

**Published:** 2023-12-14

**Authors:** Rubén Moratiel, Raúl Jimenez, Miriam Mate, Miguel Angel Ibánez, Marta M. Moreno, Ana M. Tarquis

**Affiliations:** ^1^ CEIGRAM, Universidad Politécnica de Madrid, Madrid, Spain; ^2^ AgSystems, ETSI Agronómica, Alimentaria y Biosistemas, Universidad Politécnica de Madrid, Madrid, Spain; ^3^ Entomología Aplicada a la Agricultura y la Salud, Departamento de Biotecnología Microbiana y de Plantas, Centro de Investigaciones Biológicas Margarita Salas (CIB), CSIC, Madrid, Spain; ^4^ ICEI, Universidad Complutense de Madrid, Pozuelo de Alarcón, Madrid, Spain; ^5^ Departamento Economía Agraria, Estadística y Gestión de Empresas, Escuela Técnica Superior de Ingeniería Agronómica, Alimentaria y de Biosistemas, Universidad Politécnica de Madrid (UPM), Ciudad Universitaria, Madrid, Spain; ^6^ University of Castilla-La Mancha, Higher Technical School of Agricultural Engineering in Ciudad Real, Ciudad Real, Spain; ^7^ Grupo de Sistemas Complejos, Universidad Politécnica de Madrid, Madrid, Spain

**Keywords:** photosynthesis, light intensity, light spectrum, CO_2_ concentration, net CO_2_ assimilation rate, tomato seedling

## Abstract

Artificial lighting is complementary and single-source lighting for controlled Environment Agriculture (CEA) to increase crop productivity. Installations to control CO_2_ levels and luminaires with variable spectrum and intensity are becoming increasingly common. In order to see the net assimilation of CO_2_ based on the relationship between the three factors: intensity, spectrum and CO_2_ concentration, tests are proposed on tomatoes seedling with combinations of ten spectra (100B, 80B20G, 20B80G, 100G, 80G20R, 20G80R, 100R, 80R20B, 20R80B, 37R36G27B) seven light intensities (30, 90, 200, 350, 500, 700 and 1000 μmol·m^-2^ s^-1^) and nine CO_2_ concentrations (200, 300, 400, 500, 600, 700, 800 and 900 ppm). These tomato seedlings grew under uniform conditions with no treatments applied up to the moment of measurement by a differential gas analyzer. We have developed a model to evaluate and determine under what spectrum and intensity of light photosynthesis the Net assimilation of CO_2_ (A_n_) is more significant in the leaves of tomato plants, considering the CO_2_ concentration as an independent variable in the model. The evaluation of the model parameters for each spectrum and intensity shows that the intensity has a more decisive influence on the maximum A_n_ rate than the spectra. For intensities lower than 350 μmol·m^-2^ s^-1^, it is observed that the spectrum has a greater influence on the variable A_n_. The spectra with the best behaviour were 80R20B and 80B20R, which maintained A_n_ values between 2 and 4 (μmol CO_2_·m^-2^·s^-1^) above the spectra with the worst behaviour (100G, 80G20R, 20G80R and 37B36G27R) in practically all situations. Photosynthetic Light-Use Efficiency (PLUE) was also higher for the 80B20R and 20R80B spectra with values of 36,07 and 33,84 mmol CO_2_·mol photon^-1^, respectively, for light intensities of 200 μmol·m^-2^ s^-1^ and 400 ppm of CO_2_that increased to values of 49,65 and 48,38 mmol CO_2_·mol photon^-1^ for the same light intensity and concentrations of 850 ppm. The choice of spectrum is essential, as indicated by the data from this study, to optimize the photosynthesis of the plant species grown in the plant factory where light intensities are adjusted for greater profitability.

## 1 Introduction

Light is one of the major factors that drive photosynthesis and plant development. Light spectra, intensity and duration (light dimensions) are involved in almost all vegetative processes. Among others, photomorphogenesis, phototropism, maintenance of the circadian clock or the Shade-Avoidance Syndrome (SAS) (Trojak et al., 2022). These light dimensions are also directly responsible for the efficiency of photosynthesis and determine the Net CO_2_ Assimilation Rate (A_n_). This balance fixes plants’ photo-assimilate amount and phytochemical content (Spalholz et al., 2020). Since the beginning of the century, scientific publications regarding Light Emitting Diode (LED) illumination in plants have grown exponentially, given the fine-tuning of light that new technology provides (Sipos et al., 2020). This increase manifests the amount of research performed lately, testing the effect of different dimensions of light over many crops (Viršilė et al., 2017; Sipos et al., 2020), which has been proven to be not only species- but even cultivar-dependant, each reacting differently (even though with some general similarities) to the spectra, intensity and photoperiod they were exposed (Bantis et al., 2018; Liang et al., 2021).

Artificial illumination has become relevant in the last decades as supplemental and sole-source illumination for Controlled Environment Agriculture (CEA) to increase crop productivity (Bantis et al., 2018). The recent LED technology development allows not only the reduction of costs and, therefore, the increase of the efficiency of vegetable production but also the establishment of the effect of narrow wavelength spectra over different plant processes, as mentioned above. Moreover, with LED technology, it is possible to change the most important aspects of light that affect plants: photosynthetic photon flux density (PPFD) in the photosynthetically active radiation (PAR) spectral, photoperiod, lighting mode (impulses or continuous) and light spectral composition (Berkovich et al., 2017).

In general, Red (R; 600-700 nm) and Blue (B; 400-500 nm) wavebands (RB) are the most efficient in terms of photosynthesis. They comprehended the *in vitro* absorption peaks of Chlorophyll *a* (430 nm and 662 nm) and Chlorophyll *b* (453 nm and 642 nm) when they were extracted in diethyl ether (Du et al., 1998; Pennisi et al., 2019). That is why different RB light combinations were first used as LED growing illumination (Spalholz et al., 2020; Zheng et al., 2021). Green (G; 500-600 nm) and some wavebands outside the Photosynthetically Active Radiation (PAR; 400-700 nm) range, such as Far Red (FR; 700-800 nm), have only recently started to be taken into consideration for these artificial illumination solutions since they appear to be poorly absorbed by photosynthetic pigments (Zhen et al., 2021). These authors consider that a new definition should replace the definition of PAR (400-700 nm) extended PAR (ePAR,400-750nm), which is more influential in photosynthesis and plant growth and development (Zhen et al., 2021). However, these wavebands are of importance in photosynthesis at conditions of high PPFD due to their higher transmittance within the leaves and canopy or by balancing excitation of Photosystem II (PSII) and Photosystem I (PSI) in the so-called Emmerson effect (Zhen et al., 2019). The effect of these wavelengths over plant development has shifted the light composition of artificial illumination solutions, which are starting to include broad-spectrum LEDs to cover all PAR wavebands and somehow mimic sunlight (Berkovich et al., 2017).

Being able to control the intensity and spectrum that plants receive is crucial in order to harness photosynthetic processes. It is now known that light quantity and quality have an interactive effect on photosynthesis driven by the transmittance and absorption properties of different wavelengths within the PAR spectrum (Terashima et al., 2009). Given the high absorptance of RB by the chlorophylls *in vitro*, it has been commonly accepted that they are the main drivers of photosynthesis, especially when compared to G light (van Iersel, 2017). However, this only seems true under low PPFD conditions when the photosynthetic machinery is not saturated. The low transmittance of RB light does not allow those photons to penetrate deeper leaf layers. So they are absorbed by chlorophylls even when they are already saturated, forcing them to dissipate that energy non-photochemically on the adaxial layers of the leaf. On the other hand, chlorophylls’ low absorptance of G light allows it to reach chloroplast through the whole leaf. Thus increasing the photosynthetic light use efficiency once PPFD is high enough to start saturating the upper layers of leaves (Terashima et al., 2009).

In order to dissect the interactive effect of light quality and intensity, a comprehensive study was presented quantifying the photosynthetic response of lettuce to different combinations of B, G and/or R light over a wide range of intensities (Liu and van Iersel, 2021). It was demonstrated that G photons could drive photosynthesis as efficiently as B light under low PPFD conditions. However, given their low absorptance, G light is generally less efficient in these conditions. However, at high PPFD, the photosynthetic efficiency of G light was similar to R light, not only once absorbed but on a light incident basis, with B light scoring the lowest. Similar behaviour in sunflowers on the effect of the green spectrum was reported by Terashima et al. (2009). Chlorophylls, flavonoids, and carotenoids absorb blue light, which may lead to a lesser photosynthetic yield once chlorophylls are saturated (Sun et al., 1998). This phenomenon occurs to G light on a lower basis, which might explain why R light continues to have the best behaviour. As PPFD increases, the yield for CO_2_ assimilation per photon decreases as more energy is dissipated in non-photochemical processes. However, this reduction seems slower under G light than under B or R light, assumably because of the lower absorption of green photons, thus, their better distribution throughout the leaf. This more uniform distribution reduces non-photochemical quenching (NPQ). At the same time, lower penetration of blue and red light upregulates NPQ on the upper parts of the leaves and cannot drive photosynthesis on the lower levels (Liu and van Iersel, 2021). This is important under high PPFD since NPQ is proportional to light intensity (Zhen and van Iersel, 2017).

Tomato (*Solanum lycopersicum* L.) is one of the crops most cultivated worldwide (FAOSTAT, 2022) due to its nutritional characteristics and culinary importance (Dorais et al., 2008). It is also a model plant for the study of the effect of light on plants in controlled environments, given its responsiveness to light (Yang et al., 2018). Light availability in greenhouse crops along seasons is a growing concern in northern latitudes and meridional areas such as the Mediterranean. It has been proven that supplemental LED inter-lighting illumination (R:B, 3:1) results in larger and heavier tomato fruits, especially in seasons with lower solar radiation, as well as faster fruit growth and maturation, which in turn results in higher yields (Paucek et al., 2020). This might be due to the photosynthetic capacity and light sensibility of unripped tomato fruits, which have been shown to increase their melatonin levels under RB light, a novel plant hormone that seems to promote ripening by inducing ethylene production and protect against senescence by scavenging reactive oxygen species (Li et al., 2021)

The main climate factors determining plant growth are ePAR light (Zhen et al., 2021), air temperature, air humidity, CO_2_ concentration, wind, root temperature, nutrient availability, water and oxygen. The chemical reaction of photosynthesis can be simplified as follows (Equation 1):


(1) $$light\;energy+\;6CO_{2}+12H_{2}O=C_{6}H_{12}O_{6}+6O_{2}+6H_{2}O$$

Carbon dioxide is one of the substrates for photosynthesis. Thus, it can be a limiting factor for the reaction when its concentration is below optimal. According to the Law of Minimum (also known as Liebig Law), varying only the light energy plants receive may not be enough to enhance photosynthesis properly since it is not the only substrate of the reaction. Thus, it is necessary to consider ambient CO_2_ to evaluate the photosynthetic efficiency of a given light source, adding a new dimension to the light quality and intensity interactive effect. In protected crop conditions, the environmental factors modified last are CO_2_ and lighting, the temperature and relative humidity being the first to be controlled.

In this study, we aim to identify how light intensity, its spectrum and concentration of ambient conditions of CO_2_ affect the Net CO_2_ Assimilation in tomato (*Solanum lycopersicum* L.) plants. Tomato seedlings grew under uniform conditions with no treatments applied up to the moment of measurement. Tomato leaves were exposed to spectra of different combinations of blue, green and/or red light in a wide range of intensities and increasing CO_2_ availability to assess the Net CO_2_ Assimilation under each ambient condition.

## 2 Materials and methods

### 2.1 Plant material

The trials were conducted at the Experimental Field at Agricultural Engineering School of Universidad Politécnica de Madrid (Latitude: 40.439413N; Longitude: 3.737547W) during May-Dic 2021. Tomato (*Solanum lycopersicum* L. cv. *Anairis*) seeds were sown in trays of 36 pots (3 cm length x 3 cm wide x 7 cm depth) filled with seedbed substrate with a mixture composed of 70% of white peat and 30% black peat (Tray 70/30 Gramoflor GmbH & Co. KG, Vechta, Germany) and covered with vermiculite. All plants were cultivated in a glass Greenhouse at the Experimental Field with an ACOM 2019^®^ (Acom, Balsicas, Murcia, Spain) environmental controller. The mean night/day temperature fluctuated between 18-14°C/28-20°C with adifference in day and night temperature (DIF) between +6 and +10°C and humidity between 80-60%. The maximum light intensity in the greenhouses was 400 μmolm^−2^s^−1^ (shade screens and application of calcium hydroxide, whitening, on the cover material were used) and day-night photoperiod of 14-10 h. Pots were watered daily as needed, and once a week, a general nutritive solution (5.69 mM CaNO_3_; 2.77 mM KNO_3_; 4.08 mM MgSO_4_; 1.56 mM K_2_PO_4_ and 0.048 gL^-1^ Nutrel C micronutrients Yara Inc.), was used to avoid nutrient deprivation. The conductivity of the nutrition solution was 2.1 dS·m^-1^ and a pH of 6.2. Seedlings were grown to BBCH (Biologische Bundesanstalt, Bundessortenamt und Chemische Industrie) 14-15, 4-5^th^ leaf on the main shoot unfolded (Feller et al., 1995). One day before taking the measurements, the seedlings were moved to a climatic chamber with a capacity of 350 L (Mod. Hot-Cold GL, JP Selecta, Barcelona, Spain). The conditions in the chamber were 25 °C, 80% relative humidity, and PPFD of 400 μmol/m^2^·s with the photoperiod 14-10 h day-night.

### 2.2 Carbon assimilation measurements

Tomato plants were taken for measurements 25-35 days after sowing. Only plants whose at least a fourth true leaf was completely unfolded and whose third true leaf did not show any sign of stress or deprivation were selected for analysis and discarded afterwards. Selected plants were dark-adapted for 30 minutes, and their third leaf was clipped to the leaf cuvette (PLC 3 Universal Leaf cuvette) with a window measuring 25 mm x 7 mm of a gas exchange system (CIRAS-3, PP Systems, Amesbury, MA, USA) provided with a LED Light Unit (RGBW). This dimmable light unit peaks at 446 nm (blue), 523 nm (green) and 653 nm (red) with full width at half maximum (FWHM) of 16, 36 and 17 nm, respectively (Liu and van Iersel, 2021). The combination of blue, green and red light allowed for the composition of 10 different light spectra (**Table 1**). The three monochromatic spectra of PAR radiation (100B, 100G, 100R), six combinations of binary spectra based on percentages of blue 20%, that are used in supplemental lighting (Kaiser et al., 2019) maintaining the proportions of 20%/80% of all combinations of blue, green and red spectrum and simulated natural light (reference of our study). Three plants were measured per spectrum. Each light spectrum was tested at seven different light intensities (30, 90, 200, 350, 500, 700 and 1000 μmol·m^-2^ s^-1^).

**Table 1:** Light composition of each spectrum used in the study.

Spectra	Fraction of photon flux (%)		
	Blue	Green	Red
100B	100	0	0
80B20G	80	20	0
20B80G	20	80	0
100G	0	100	0
80G20R	0	80	20
20G80R	0	20	80
100R	0	0	100
80R20B	20	0	80
20R80B	80	0	20
37R36G27B	37	36	27

**Figure 1:**
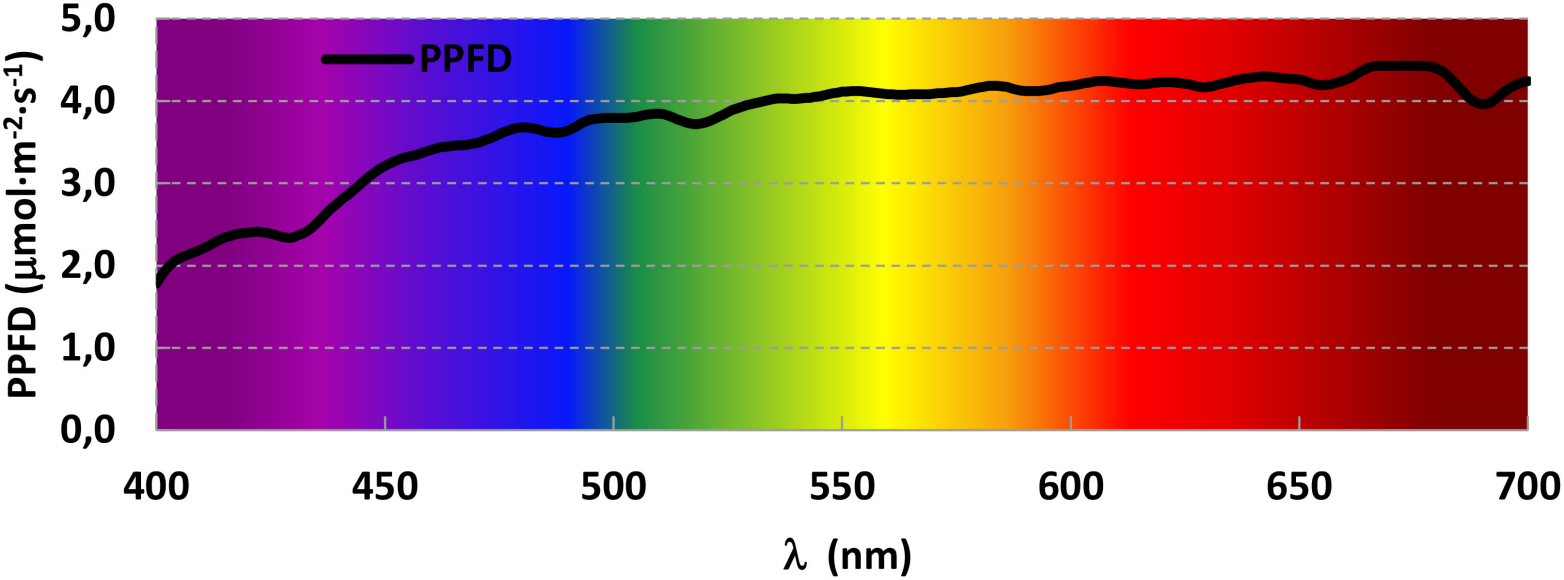
Averaged solar radiation in the interval of photosynthetic active radiation (PAR) during a sunny summer day (August 6, 2021) in the Experimental Field in Madrid, Spain (Latitude: 40.439413 N, Longitude: 3.737547W).

Different spectra were designed so it would be possible to determine the effect of each monochromatic light as well as their interaction by pairs. A trichromatic spectrum was designed to average the light a plant would receive on a sunny summer day. Therefore, solar radiation was recorded in triplicate at three different moments of a sunny summer day (morning, noon and evening) using a spectroradiometer (PN-200, UPRtek, Zhunan Township, Miaoli County, Taiwan) and those nine readings were averaged. The resulting spectrum was then divided into segments of 100 nm, and the fraction Blue (400-499 nm), Green (500-599 nm) and Red (600-699 nm) was calculated and used to design the trichromatic spectrum (**Figure 1**).

To study the photosynthesis efficiency under different spectra, intensities, and CO_2_ concentrations, we constructed CO_2_ response curves for each intensity and spectrum using a Rapid A/Ci Response (RACiR) technique (Saathoff and Welles, 2021). The photosynthetic light-use efficiency (PLUE) was calculated, which is defined as the slope between the net CO_2_ assimilation rate (A_n_) and incident PPFD on the leaf.

After 5 minutes of acclimatization in the lowest CO_2_ concentration and light intensity (200 ppm CO_2_, 30 µmol·m^-2^·s^-1^ photons), three Net CO_2_ Assimilation rates (A_n_), Stomatic Conductance, Vapour Pressure Deficit (VPD) and Water Use Efficiency (WUE) readings were taken at a 10 seconds interval. CO_2_ concentration was then raised to 100 ppm, and the leaf was kept in these conditions for two minutes before recording the three readings. This continued through all the CO_2_ concentrations studied (200, 300, 400, 500, 600, 700, 800 and 900 ppm). Once the maximum concentration is reached, the light intensity rises to the next lowest intensity of the study. CO_2_ concentration then decreases by 100 ppm per triplicate of readings until the lowest concentration is reached, and then light intensity rises again. This process is repeated until all light intensities (30, 90, 200,350, 500, 700 and 1000 µmol·m^-2^·s^-1^) are reached. Recordings are taken for every CO_2_ concentration and light intensity in the study (**Table 2**). Environmental conditions inside the cuvette were controlled by the leaf gas exchange system setting values of leaf temperature of 25.0 ± 0.4°C and VPD of 1.6 ± 0.3kPa.

**Table 2:** Environmental conditions (CO_2_ Concentration and Light Intensity) were set for each set of three readings (N) during measurements.

N	[CO_2_] (ppm)	I (µmol·m^-2^·s^-1^)	N	[CO_2_] (ppm)	I (µmol·m^-2^·s^-1^)	N	[CO_2_] (ppm)	I (µmol·m^-2^·s^-1^)
1	200	30	20	500	200	39	800	500
2	300	30	21	600	200	40	900	500
3	400	30	22	700	200	41	900	700
4	500	30	23	800	200	42	800	700
5	600	30	24	900	200	43	700	700
6	700	30	25	900	350	44	600	700
7	800	30	26	800	350	45	500	700
8	900	30	27	700	350	46	400	700
9	900	90	28	600	350	47	300	700
10	800	90	29	500	350	48	200	700
11	700	90	30	400	350	49	200	1000
12	600	90	31	300	350	50	300	1000
13	500	90	32	200	350	51	400	1000
14	400	90	33	200	500	52	500	1000
15	300	90	34	300	500	53	600	1000
16	200	90	35	400	500	54	700	1000
17	200	200	36	500	500	55	800	1000
18	300	200	37	600	500	56	900	1000
19	400	200	38	700	500			

This has been performed over three plants per spectrum described in **Table 1**.

### 2.3 Statistical analysis

A nonlinear mixed effects model (Lindstrom and Bates, 1990) was estimated to relate assimilation rate as a response variable and light spectra, light intensity and CO_2_ concentration levels as explanatory variables.

An asymptotic regression model was used to describe limited growth, where the response variable approaches a horizontal asymptote as CO_2_ approaches infinity.

The model used was:


(2) $$A_{n}=c+\left(d-c\right)\times \left(1-e^{-\frac{CO_{2}}{b}}\right)$$

Where

*A_n_* is the Net CO_2_ Assimilation rate, *c* is the value of A_n_ when the CO_2_ level is zero, *d* is the maximum attainable A_n_, 1/b is proportional to A_n_’s relative rate of increase as CO_2_ increases, and *e* is a random error term. This term (*e*) was assumed to have a normal distribution with zero mean and different variance for each intensity level.

It is assumed that the values of c, d and e depend on the light spectra and intensity levels.

c = Intensity + Spectra

d = Intensity + Spectra + *u*

b = Intensity + Spectra

where *u* is a random term that considers the variability for each plant in the parameter d. The random term *u* was assumed to have a normal distribution with mean 0 and variance $\sigma_{u}^{2}$ .

The statistical model, as defined, took into account the hierarchical structure in which the data was obtained: Three plants per spectrum were measured, and each plant was tested at different light intensities and CO_2_ concentrations. The experimental data estimated the parameters b, c and d based on the intensity and spectrum levels used. Hypothesis tests were performed to determine significant differences between their estimates and standard errors. Normality assumptions were also checked using the residuals of the estimated model. The bigger the *b* parameter, the lower the curvature; hence, the higher the theoretical CO_2_ saturation point. The more intensity applied, the higher the *d* parameter and the highest theoretical maximum A_n_ is reached. This model studies the effect of the different spectra and intensities over the *c*, *d* and *b* parameters.

Statistical analysis was performed in the R environment (R Core Team, 2021). The model estimation was done with the *nlme* package (Pinheiro et al., 2021), a testing hypothesis was carried out with the *emmeans* package (Russell, 2022) and graphics with the ggplot package (Wickham, 2009).

## 3 Results

### 3.1 Changes in net carbon assimilation due to varying CO_2_ concentration, light intensity and spectra used

**Figure 2:**
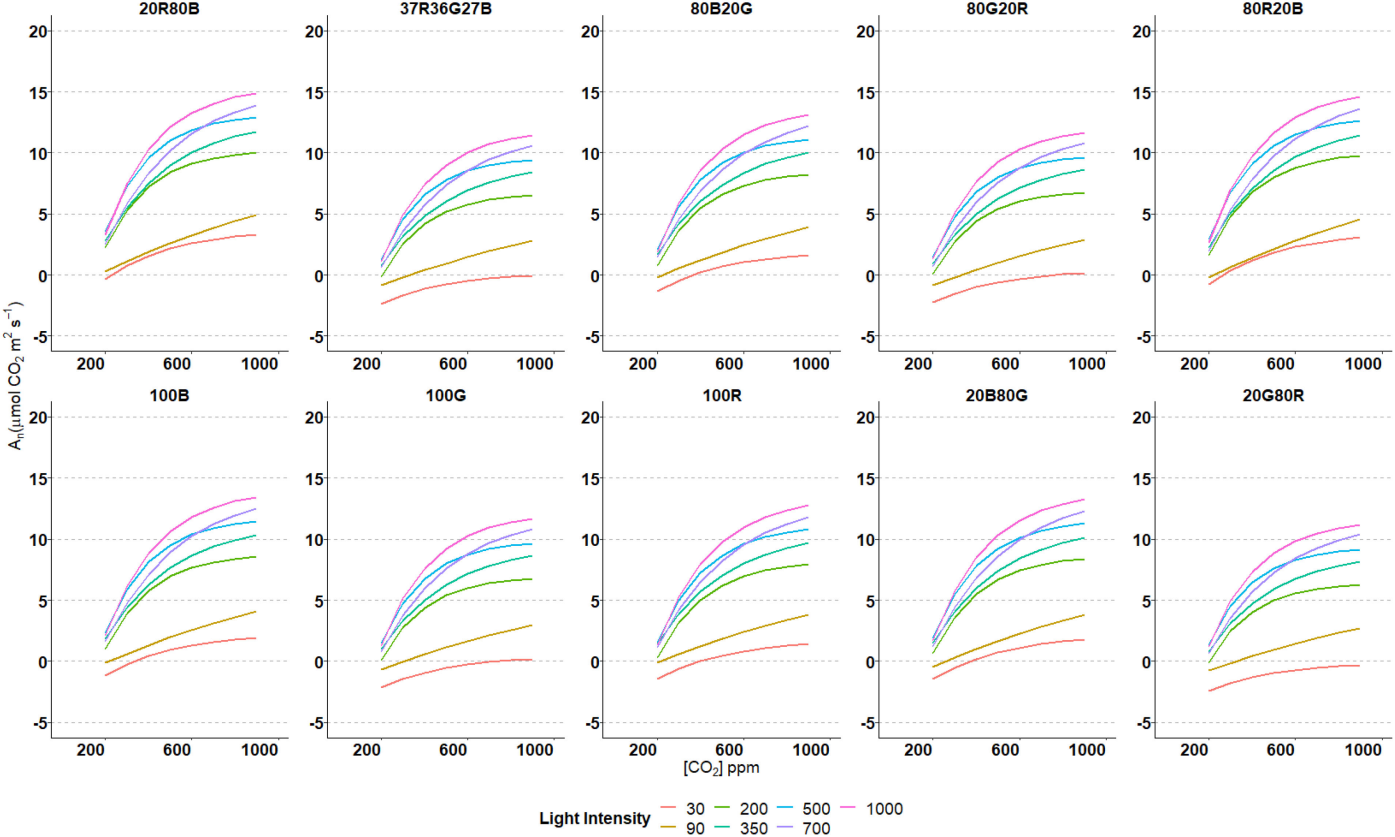
Estimated nonlinear regression models for A_n_ and CO_2_ concentrations at different intensities and spectrums used.

Net Carbon Assimilation (A_n_) was assessed at eight different CO_2_ concentrations for seven light intensity values at ten light spectra varying R, G and B light fractions (**Figure 2**) on the third true leaf of tomato plants. For every spectrum, at light intensities of 200 µmol·m^-2^·s^-1^ or higher, A_n_/CO_2_-concentration response showed the typical display of an asymptotic curve, A_n_ rising rapidly as CO_2_ increased at lower levels until reaching a concentration in which A_n_ increase slows down and even stops going up. The higher the intensity, the higher the curvature, reaching higher A_n_ values in all spectra. At lower light intensities (30 and 90 µmol·m^-2^·s^-1^), CO_2_ response curves were more lineal, not showing a pronounced change in the tendency of the curve. The curves’ shapes were similar at all the spectra and intensities used, pointing out the same A_n_ behaviour due to increases in CO_2_ concentration. However, the absolute values of A_n_ changed through different spectra. The highest A_n_ values at every light intensity were observed at 20R80B and 80R20B spectrums. The lowest A_n_ values were archived by the 20G80R spectrum, followed by the trichromatic spectrum 37R36G27B (**Figure 2**). The highest A_n_ values, 18.9 μmol CO_2_·m^-2^·s^1^, were obtained at CO_2_ concentrations of 700, 800 and 900 ppm, and with 1000 µmol·m^-2^·s^-1^ light intensity and in 80R20B spectrum. Contrary, the A_n_ lowest values, -4.9 and -3.2 μmol CO_2_·m^-2^·s^-1^, were reached in 100 G and 20G80R spectrums, and CO_2_ intensities of 30 µmol·m^-2^·s^-1^ and 200 ppm, respectively.

### 3.2 Model

The most frequently used methods to understand how C_3_ plant photosynthesis responds to changes in CO_2_ concentration are based on the studies of Farquhar et al. (1980). These biochemical models focus on the activity of ribulose 1:5 bisphosphate carboxylase/oxygenase (Rubisco). We have developed a model to determine under which light photosynthesis spectrum and intensity is greater for tomato plants’ leaves, considering the concentration of CO_2_ as an independent variable.

**Table 3** studies the interference of the model with the intensity of illumination. The simulated solar spectrum of 37R36G27B is a reference for the analysis. The intensity of 350 μmol·m^-2^·s^-1^ is used as a reference to analyze the spectra (**Table 4**). The same trend is observed in each spectrum or intensity compared. It shows an increase or decrease of the parameters by the same amount (**Table 5**).

**Table 3:** Mean parameters *d* and *b* values from the model (Equation 2) for every intensity examined in the spectrum 37R36G27B.

Intensity (μmol·m^-2^·s^-1^)	*d* ( ± s.e.)		*b* ( ± s.e.)	
30	0.26^a^	± 0.42	320.71^abc^	± 43.90
200	6.74^bcd^	± 0.39	202.98^abc^	± 8.13
90	7.73^bcd^	± 1.50	1264.96^d^	± 249.9
350	9.28^c^	± 0.42	310.32^c^	± 16.08
500	9.68^c^	± 0.39	202.34^a^	± 7.83
1000	11.98^d^	± 0.4	231.78^b^	± 8.61
700	12.01^d^	± 0.4	333.32^c^	± 17.34

Mean values ± standard error. Mean values that include a common letter in the same column are not statistically different (p ≤ 0.05).

**Table 4:** Mean ± values of parameters *d* and *b* from the model (Equation 2) for every spectrum light intensity of 350 μmol·m^-2^·s^-1^.

Spectra	*d* ( ± s.e.)		*b* ( ± s.e.)	
80R20G	8.96^a^	± 0.42	305.77^a^	± 16.07
37R36G27B	9.28^a^	± 0.42	310.32^a^	± 16.08
20R80G	9.49^ab^	± 0.42	308.37^a^	± 16.04
100G	9.51^ab^	± 0.42	310.90^a^	± 16.03
100R	10.85^ab^	± 0.42	334.68^b^	± 16.21
20G80B	11.06^cd^	± 0.42	321.73^ab^	± 16.06
80G20B	11.27^cd^	± 0.42	325.59^ab^	± 16.02
100B	11.41^cd^	± 0.42	320.69^ab^	± 16.02
80R20B	12.63^de^	± 0.43	321.12^ab^	± 15.91
20R80B	12.82^e^	± 0.42	314.45^ab^	± 15.88

Mean values ± standard error (s.e.). Mean values that include a common letter in the same column are not statistically different (p ≤ 0.05).

**Table 5:** Values of parameters *d*, *b* and *c* from the model of Equation 2 for every spectrum and intensity tested.

Spectra	Parameter	Intensities (μmol·m^-2^·s^-1^)							▵
		30	90	200	350	500	700	1000	
**37R36G27B**	***d***	0.26	7.73	6.74	9.28	9.68	12.01	11.98	-
	***b***	320.71	1264.96	202.98	310.32	202.34	333.32	231.78	-
	***c***	-4.62	-2.34	-11.61	-6.89	-12.88	-8.76	-13.74	-
**100B**	***d***	2.39	9.86	8.87	11.41	11.81	14.14	14.11	2.13
	***b***	331.08	1275.33	213.35	320.69	212.71	343.69	242.15	10.37
	***c***	-4.12	-1.84	-11.11	-6.39	-12.39	-8.26	-13.24	0.50
**100G**	***d***	0.49	7.96	6.97	9.51	9.91	12.24	12.21	0.23
	***b***	321.3	1265.55	203.57	310.91	202.93	333.91	232.37	0.59
	***c***	-4.45	-2.17	-11.45	-6.73	-12.72	-8.60	-13.57	0.17
**100R**	***d***	1.83	9.3	8.31	10.85	11.25	13.58	13.55	1.57
	***b***	345.07	1289.32	227.34	334.68	226.7	357.68	256.14	24.36
	***c***	-3.97	-1.69	-10.96	-6.24	-12.23	-8.11	-13.09	0.65
**20B80G**	***d***	2.25	9.72	8.73	11.27	11.67	14.00	13.97	1.99
	***b***	335.98	1280.23	218.25	325.59	217.61	348.59	247.05	15.27
	***c***	-4.50	-2.22	-11.49	-6.77	-12.76	-8.64	-13.62	0.12
**20G80R**	***d***	-0.06	7.41	6.42	8.96	9.36	11.69	11.66	-0.32
	***b***	316.17	1260.42	198.44	305.78	197.8	328.78	227.24	-4.54
	***c***	-4.47	-2.19	-11.46	-6.74	-12.73	-8.61	-13.59	0.15
**20R80B**	***d***	3.8	11.27	10.28	12.82	13.22	15.55	15.52	3.54
	***b***	324.85	1269.1	207.12	314.46	206.48	337.46	235.92	4.14
	***c***	-3.88	-1.60	-10.87	-6.15	-12.14	-8.02	-13.00	0.74
**80B20G**	***d***	2.04	9.51	8.52	11.06	11.46	13.79	13.76	1.78
	***b***	332.13	1276.38	214.4	321.74	213.76	344.74	243.2	11.42
	***c***	-4.11	-3.60	-3.09	-2.58	-2.07	-1.56	-1.05	0.51
**80G20R**	***d***	0.47	7.94	6.95	9.49	9.89	12.22	12.19	0.21
	***b***	318.76	1263.01	201.03	308.37	200.39	331.37	229.83	-1.95
		-4.63	-4.64	-4.65	-4.66	-4.67	-4.68	-4.69	-0.01
**80R20B**	***d***	3.6	11.07	10.08	12.62	13.02	15.35	15.32	3.34
	***b***	331.52	1275.77	213.79	321.13	213.15	344.13	242.59	10.81
	***c***	-4.44	-2.16	-11.43	-6.71	-12.70	-8.58	-13.56	0.18

Δ show the change rate of that parameter with baseline 37R36G27B spectrum and 350 μmol·m^-2^·s^-1^.

In the analyses carried out in the model, one of the most important parameters is to determine *d* (asymptotic value of maximum A_n_ when the CO_2_ concentration tends to infinity), with a higher value of *d*, higher production potential. **Table 3** shows the estimated values of *d* for each lighting intensity level. It is observed that there is a positive relationship between the intensity and the values of d. The increase in intensity tends to increase the estimated value of the parameter *d*. The highest intensities, 700 and 1000 μmol·m^-2^·s^-1^, show the highest values of parameter d (12.01 and 11.98, respectively), showing significant differences for the other intensities. This trend would be observed regardless of the spectrum used, decreasing or increasing the estimated values by the same amount depending on the spectrum used. The estimated values of parameter *b* (responsible for curvature) fluctuate between 202.34 for 500 μmol·m^-2^·s^-1^ and 1264.96 for 90 μmol·m^-2^·s^-1^ (**Table 3**). Note that all the intensities, except for 90 μmol·m^-2^·s^-1^, are between 200 and 340. For intensity of 90 μmol·m^-2^·s^-1^, very high *b* values are observed, indicating that it practically approaches its maximum linearly. At higher values of *b*, the curve tends to be more linear and needs higher levels of CO_2_ to reach its maximum asymptotic value. It is observed that the *b* values do not follow an intensity pattern. However, at low intensities (30 and 90 μmol·m^-2^·s^-1^), this parameter shows more significant fluctuations, as the standard error values point out, being much higher than those of the higher intensities (**Table 3**).

**Table 4** shows the model’s behaviour depending on the light spectrum for an intensity of 350 μmol·m^-2^·s^-1^. As a function of the spectrum, the d and b parameters range values are 8.96 to 12.82 and 305.77 to 334.68, respectively. These values are significantly lower than those required by the light intensity (*d* from 0.26 to 12.01 and *b* from 202.34 to 1264.96). It is observed how the spectra 80R20B and 20R80B are the ones that would reach the highest potential values of A_n_, with significant differences concerning the other spectra. The spectrum that reaches the lowest maximum A_n_ are 20G80R, 37R36G27B, 80G20R, 100G and 100R, with no significant differences (**Table 4**). Parameter *b* is a parameter with few fluctuations due to the spectra, with no significant differences between 20G80R, 37R36G27B, 80G20R, 100G 80B20G, 20B80G, 100B, 80R20B and 20R80B. In addition, another group is formed by 80B20G, 20B80G, 100B, 80R20B, 20R80B and 100R without significant differences.

**Table 5** shows the model parameter values (*d*, *b and c)* for each light intensity and spectrum used in this experiment. Trichromatic spectrum 37R36G27B at 350 μmol·m^-2^·s^-1^ has been chosen as a reference since it was designed as sunlight radiation. Its values have been used as the baseline. The curve can be obtained for each intensity and spectrum in **Table 5**.

**Figure 3:**
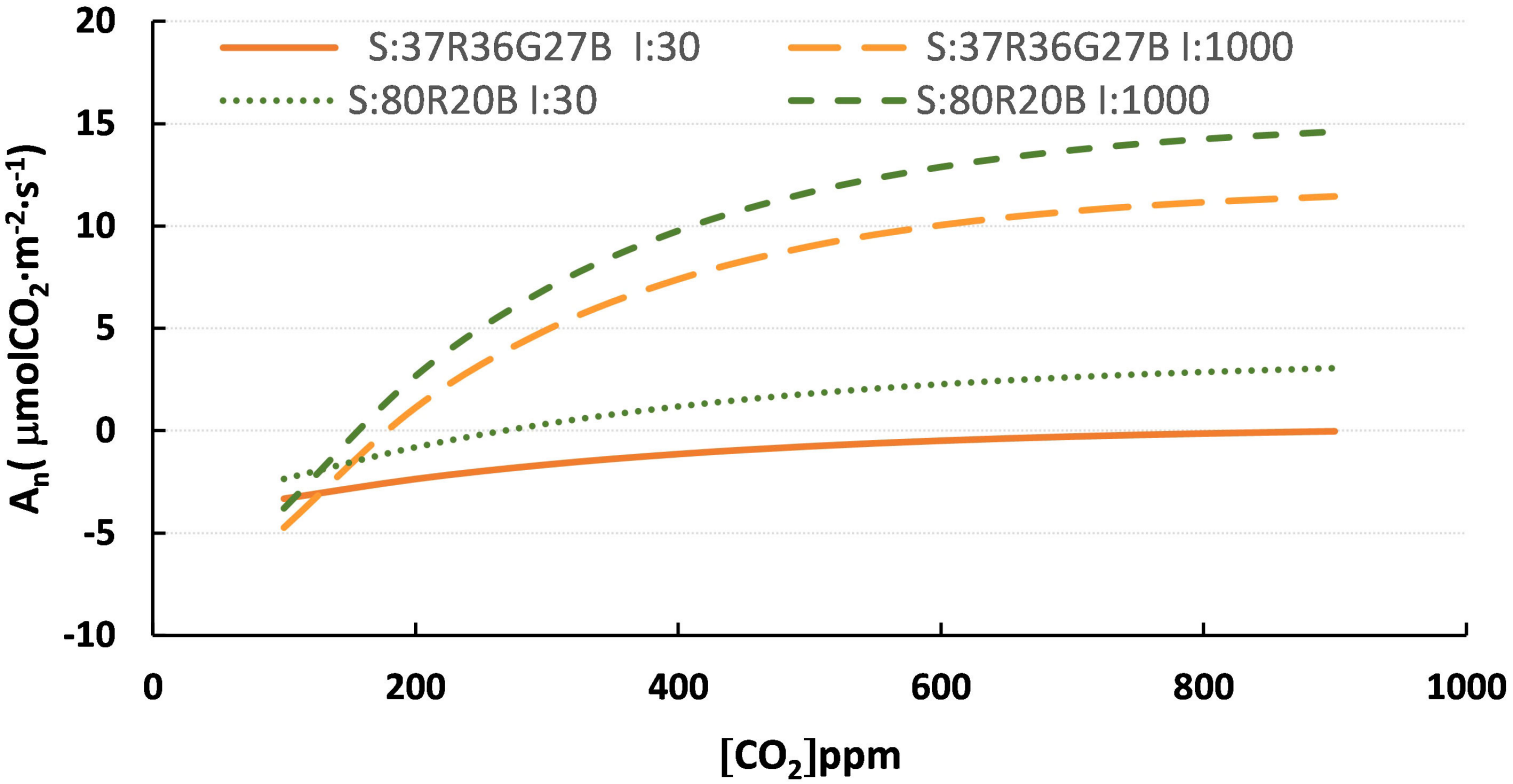
Curves of the spectra model 80R20B and R37G36B27 and PPFD of 30 and 1000 μmol·m^-2^·s^-1^. S:R37G36B27 I:30, spectra R37G36B27 at 30 μmol·m^-2^·s^-1^; S:R37G36B27 I:1000, spectra R37G36B27 at 1000 μmol·m^-2^·s^-1^; S: 80R20B I:30, spectra 80R20B at 30 μmol·m^-2^·s^-1^ and S:80R20B I:1000, spectra 80R20B at 1000 μmol·m^-2^·s^-1^.

**Figure 3** compares the models with two PPFDs and two spectra. It is observed how the -PPFD component influences more than the spectra. However, the spectra show different trends with the same intensity, observing differences in A_n_ among them.

When applying values from **Table 5** to Equation 2. values for A_n_ can be calculated for each intensity and spectrum for any fixed CO_2_ concentration (**Table 6**). This work is particularised for three possible scenarios of CO_2_ concentration taken into consideration based on different real-life scenarios that can occur under a greenhouse (Both et al., 2017). The first scenario is the study of the A_n_ of the spectra for the atmospheric concentration (400 ppm), and the second case is the increase in carbon fertilization up to levels of 850 ppm, a situation that can be frequently reached in the carbon fertilization of greenhouses of crops of C_3_ metabolism like rose and tomato. The last scenario is the reduction of the CO_2_ concentration to levels of 200 ppm, a situation that can occur at certain times of the day with poor ventilation in greenhouses and a high rate of photosynthesis in crops with high LAI (Leaf Area Index).

**Table 6:** Calculated values (according to **Table 5** and Equation 2) of A_n_ (μmol CO_2_·m^-2^·s^-1^) for each light intensity and spectrum tested at three CO_2_ concentration scenarios: 400 ppm as atmospheric CO_2_ concentration, 850 ppm as carbon- fertilized greenhouse concentration, and 200 ppm as the case of a CO_2_-deprived ambient due to a high photosynthetic rate.

Spectra	Intensity light (incident PPFD, μmol·m^-2^·s^-1^)																				
	30	90	200	350	500	700	1000	30	90	200	350	500	700	1000	30	90	200	350	500	700	1000
	200 ppm CO_2_							400 ppm CO_2_							850 ppm CO_2_						
**37R36G27B**	-2,36	-0,87	-0,11	0,79	1,28	0,61	1,13	-1,14	0,39	4,18	4,82	6,56	5,75	7,40	-0,08	2,59	6,46	8,23	9,34	10,39	11,32
**100B**	-1,17	-0,14	1,05	1,87	2,36	1,62	2,14	0,45	1,31	5,81	6,30	8,12	7,14	8,87	1,89	3,85	8,50	10,15	11,37	12,25	13,29
**100G**	-2,16	-0,69	0,07	0,97	1,46	0,79	1,31	-0,93	0,58	4,39	5,02	6,76	5,95	7,60	0,14	2,78	6,69	8,45	9,57	10,61	11,55
**100R**	-1,42	-0,11	0,32	1,44	1,53	1,18	1,35	0,01	1,24	4,99	5,67	7,23	6,49	7,96	1,34	3,62	7,85	9,50	10,70	11,57	12,59
**20B80G**	-1,47	-0,49	0,64	1,51	1,93	1,24	1,69	0,20	0,98	5,50	5,99	7,78	6,81	8,50	1,71	3,57	8,32	9,94	11,18	12,02	13,08
**20G80R**	-2,40	-0,77	-0,10	0,80	1,32	0,64	1,19	-1,30	0,43	4,04	4,72	6,44	5,68	7,32	-0,36	2,52	6,17	7,99	9,06	10,16	11,06
**20R80B**	-0,35	0,29	2,23	2,78	3,59	2,52	3,31	1,56	1,89	7,21	7,50	9,57	8,35	10,29	3,24	4,69	9,93	11,55	12,81	13,65	14,74
**80B20G**	-1,33	-0,18	0,80	1,69	2,11	1,45	1,91	0,20	1,23	5,48	6,03	7,79	6,88	8,55	1,56	3,69	8,15	9,82	11,01	11,92	12,94
**80G20R**	-2,25	-0,84	0,08	0,92	1,49	0,74	1,32	-0,98	0,44	4,41	5,01	6,79	5,94	7,64	0,12	2,69	6,68	8,45	9,56	10,61	11,55
**80R20B**	-0,80	-0,23	1,64	2,25	2,96	1,97	2,66	1,19	1,41	6,77	7,06	9,08	7,87	9,77	2,98	4,28	9,68	11,25	12,54	13,33	14,45

Blue-containing spectra show higher A_n_ values than their Red and Green counterparts, followed by red-containing spectra. G light seems to have a lower effect in enhancing Net Carbon Assimilation. The highest values for A_n_ are archived by the 20R80B spectrum, followed by the 80R20B spectrum and then by the monochromatic 100B compared to other spectra at the same light intensity and CO_2_ concentrations. The lowest A_n_ values belong to the 20G80R spectrum, followed by the trichromatic 37R36G27B. **Table 6** shows that under conditions of low CO_2_ concentration (200 ppm), the A_n_ values begin to be positive at incident PPFD of 200 μmol·m^-2^·s^-1^, although spectra such as 20R80B take positive values at 90 μmol·m^-2^·s^-1^. The A_n_ values do not exceed 4 μmolCO_2_·m^-2^·s^-1^ at these CO_2_ concentrations and any PPFD. The highest values are reached in the 80R20B and the 20R80B spectra (2.66 and 3.31, respectively).

For values of 400 ppm of CO_2_, even at intensities of 30 μmol·m^-2^·s^-1^, positive A_n_ values are observed for all spectra except for 37R36G27B, 100G and 20G80R. For concentrations of 400 ppm of CO_2_ with PPFD of 350 μmol·m^-2^·s^-1^, the spectra that reached 7 μmol CO_2_·m^-2^·s^-1^ were 80R20B and 20R80B. The same trend is obtained for these two spectra at concentrations of 850 ppm of CO_2_ and 350 μmol·m^-2^·s^-1^ of PPFD, where they are the only ones that reach 11 μmolCO_2_·m^-2^·s^-1^ of A_n_.

In **Table 7**, a relative comparison is made taking as reference the A_n_ of 350 μmol·m^-2^·s^-1^, with 400 ppm of CO_2_ and spectrum of 37R36G27B (with a value of 4.82 μmolCO_2_·m^-2^·s^-1^) and determined the percentages related to this situation Equation 3. The values shown result from the value obtained as a reference minus the value divided by the reference and multiplied by 100. In this case, it can be seen how the values of the 20R80B and 80R20B spectra are always higher than the reference and other spectra, although it will depend on the PPFD and the CO_2_ concentration. The 20R80B and 80R20B spectra with a lower light intensity of 150 μmol·m^-2^·s^-1^ than the reference (reference with 350 μmol·m^-2^·s^-1^ and type of spectra with 200 μmol·m^-2^·s^-1^) show values of A_n_ that are 50 and 40% higher, respectively.

**Table 7:** Calculated A_n_ increment relative to that of the spectrum 37R36G27B (designed after sun radiation) at 350 µmol·m^-2^·s^-1^ and 400 ppm of CO_2_ (yellow cell) for every spectrum and intensity tested in the three theoretical CO_2_ concentration scenarios of 200 ppm, 400 ppm and 700 ppm.

**Spectra**	**Intensity light (incident PPFD, μmol·m^-2^·s^-1^)**																				
	**30**	**90**	**200**	**350**	**500**	**700**	**1000**	**30**	**90**	**200**	**350**	**500**	**700**	**1000**	**30**	**90**	**200**	**350**	**500**	**700**	**1000**
	**200 ppm CO_2_**							**400 ppm CO_2_**							**850 ppm CO_2_**						
**37R36G27B**	-149	-118	-102	-84	-73	-87	-77	-124	-92	-13	0	36	19	53	-102	-46	34	71	94	115	135
**100B**	-124	-103	-78	-61	-51	-66	-56	-91	-73	20	31	68	48	84	-61	-20	76	110	136	154	176
**100G**	-145	-114	-98	-80	-70	-84	-73	-119	-88	-9	4	40	23	58	-97	-42	39	75	98	120	139
**100R**	-129	-102	-93	-70	-68	-76	-72	-100	-74	3	18	50	35	65	-72	-25	63	97	122	140	161
**20B80G**	-131	-110	-87	-69	-60	-74	-65	-96	-80	14	24	61	41	76	-65	-26	72	106	132	149	171
**20G80R**	-150	-116	-102	-83	-73	-87	-75	-127	-91	-16	-2	33	18	52	-107	-48	28	66	88	111	129
**20R80B**	-107	-94	-54	-42	-26	-48	-31	-68	-61	50	56	98	73	113	-33	-3	106	139	165	183	206
**80B20G**	-128	-104	-83	-65	-56	-70	-61	-96	-75	14	25	62	43	77	-68	-24	69	104	128	147	168
**80G20R**	-147	-117	-98	-81	-69	-85	-73	-120	-91	-9	4	41	23	58	-98	-44	38	75	98	120	139
**80R20B**	-117	-105	-66	-53	-39	-59	-45	-75	-71	40	46	88	63	102	-38	-11	101	133	160	176	200

▬ A_n_ <-99 ▬ -99< A_n_ <-66 ▬ -66< A_n_ <-33 ▬ -33< A_n_ <33 ▬ 33< A_n_ <66 ▬ 66> A_n_ <99 ▬ 99< A_n_

The font number shows whether the calculated A_n_ is higher (in green) or lower (in red) than the reference. The background darkness indicates whether the An value is higher (in green) or lower (in red) than that of 37R36G27B at 350 µmol·m^-2^·s^-1^ at the same CO_2_ concentration.


$$A_{n\;relative}=100*\left(\frac{A_{n\;i}-A_{n\;reference}}{A_{n\;reference}}\right)\;\;\;\;\;\;\left[3\right]$$

Where

A_n reference_ = Value of A_n_ with spectrum 37R36G27B with a PPFD of 350 μmol·m^-2^·s^-1^ and CO_2_ concentration of 400 ppm. A_ni_ = Value of A_n_ with spectra, PPFD and CO_2_ concentrations selected according to **Table 6**.

Although the relative increases in A_n_ are marked mainly by the intensity of light and the concentration of CO_2_. **Table 7** shows the spectra’s influence on the Net Carbon Assimilation. Values in A_n_ with PPFD conditions of 1000 μmol·m^-2^·s^-1^ and 400 ppm of CO_2_ in the 37R36G27B spectrum are similar to those obtained by the 20R80B and 80R20B spectra at PPFD of 350 μmol·m^-2^·s^-1^ with 400 ppm of CO_2_.

**Table 8** shows how the variable PLUE changes depending on the spectrum, intensity, and concentration of CO_2_. It is observed that PLUE increases as the concentration of CO_2_ increases analyzed. At low concentrations of CO_2_ (200 ppm), the highest values of PLUE occur at intensities of 350 μmol·m^-2^·s^-1^, while as the concentration of CO_2_ increases, the highest efficiency is reached at values of 90 -200 μmol·m^-2^·s^-1^. Concerning the spectra, although all of them follow the same behaviour, there are differences between them. The ones that show the best efficiency are the spectrum of 20R80B and 20B80R. Concerning light intensity, maximum PLUE values are shown for all spectra and with 200 ppm CO_2_ in values around 200-350 PPFD, as we increase CO_2_ to 400 and 850 ppm, the maximum PLUE values drop to 200 and 200-90 PPFD, respectively **Table 6**. Calculated values (according to **Table 5** and Equation 2) of A_n_ (μmol CO_2_·m^-2^·s^-1^) for each light intensity and spectrum tested at three CO_2_ concentration scenarios: 400 ppm as atmospheric CO_2_ concentration, 850 ppm as carbon- fertilized greenhouse concentration, and 200 ppm as the case of a CO_2_-deprived ambient due to a high photosynthetic rate.

**Table 8:** Photosynthetic Light-Use Efficiency (PLUE, mmol CO_2_/mol photon) for each light intensity and spectrum tested at three CO_2_ concentration scenarios: 400 ppm as atmospheric CO2 concentration, 850 ppm as the concentration of a carbon fertilized greenhouse and 200 ppm as the case of a CO_2_ deprived ambient due to a high photosynthetic rate.

Spectra	Intensity light (incident PPFD, μmol·m^-2^·s^-1^)																				
	200 ppm CO_2_							400 ppm CO_2_							850 ppm CO_2_						
	30	90	200	350	500	700	1000	30	90	200	350	500	700	1000	30	90	200	350	500	700	1000
**37R36G27B**	-78,52	-9,64	-0,55	2,26	2,57	0,87	1,13	-38,07	4,33	20,91	13,78	13,11	8,22	7,40	-2,82	28,75	32,31	23,53	18,68	14,84	11,32
**100B**	-38,94	-1,58	5,23	5,34	4,72	2,32	2,14	14,84	14,55	29,03	17,99	16,24	10,21	8,87	63,01	42,80	42,49	29,01	22,73	17,50	13,29
**100G**	-72,03	-7,66	0,37	2,79	2,93	1,13	1,31	-31,08	6,39	21,94	14,35	13,52	8,50	7,60	4,65	30,94	33,43	24,16	19,13	15,15	11,55
**100R**	-47,29	-1,23	1,58	4,12	3,06	1,69	1,35	0,34	13,79	24,96	16,21	14,45	9,27	7,96	44,54	40,17	39,26	27,15	21,39	16,52	12,59
**20B80G**	-49,07	-5,48	3,21	4,31	3,85	1,78	1,69	6,59	10,93	27,48	17,11	15,57	9,73	8,50	57,08	39,70	41,59	28,41	22,36	17,18	13,08
**20G80R**	-80,09	-8,59	-0,51	2,28	2,65	0,92	1,19	-43,48	4,75	20,20	13,47	12,87	8,11	7,32	-11,99	28,05	30,87	22,82	18,12	14,51	11,06
**20R80B**	-11,64	3,17	11,14	7,94	7,19	3,60	3,31	51,94	20,96	36,07	21,44	19,13	11,92	10,29	107,97	52,09	49,65	33,00	25,61	19,50	14,74
**80B20G**	-44,26	-1,96	4,02	4,84	4,22	2,07	1,91	6,52	13,65	27,42	17,23	15,58	9,83	8,55	52,14	40,99	40,74	28,05	22,03	17,03	12,94
**80G20R**	-75,11	-9,37	0,42	2,63	2,98	1,06	1,32	-32,80	4,93	22,05	14,32	13,59	8,49	7,64	3,85	29,89	33,40	24,14	19,12	15,15	11,55
**80R20B**	-26,60	-2,58	8,20	6,43	5,91	2,81	2,66	25,12	15,65	33,84	20,16	18,16	11,24	9,77	99,36	47,55	48,38	32,14	25,09	19,04	14,45
**Mean**	-52,36	-4,49	3,31	4,29	4,01	1,82	1,80	-4,01	10,99	26,39	16,61	15,22	9,55	8,39	41,78	38,09	39,21	27,24	21,43	16,64	12,66

## 4 Discussion

Since McCree’s work (McCree, 1971), Red and Blue light have been considered the most efficient wavebands for photosynthesis. This correlates with chlorophyll absorption spectra, which peak at about 430 and 660 nm (Viršilė et al., 2017). In the literature, no references have been found that deal jointly with the combination of the three factors of light intensity, spectrum and CO_2_ concentrations of the photosynthetic responses of seedlings grown under the same conditions until measurements with spectrum change. Other authors studied plants grown in different conditions from the beginning of their growth. Authors such as Huber et al. (2021) studied the relationship between light intensities (three daily light integral, DLIs) and three different CO_2_ concentrations but with a fixed spectrum ratio of 40B:60R. Other authors focus on the relationship between light intensities and light quality in spectra of red and blue combinations (Hernández and Kubota, 2012; Zheng et al., 2021).

In our tests, we have observed (**Tables 3**, **4**) that the influence of intensity on parameter *d* (asymptotic value of maximum A_n_ when the CO_2_ concentration tends to infinity) is higher than the effect of the tested spectra. The net assimilation rate (A_n_) obtained in the trial was around between 11-15 μmol CO_2_·m^-2^·s^-1^ for 1000 μmol·m^-2^·s^-1^ of PPFD. These values agree with those obtained by Yang et al. (2018) for tomato seedlings at 6-leaf stage. In our model, the *d* parameter values, when the intensities of 30 and 700 μmol·m^-2^·s^-1^, is 11.75 while the fluctuation of *d* as a spectrum function is 3.86. The effects of light intensity or PPFD is the primary variable to identify in the light needs of plants (DLI). Usually, increases in light intensity correlate with increases in net photosynthesis rate (A_n_) (Bowes et al., 1972; Fan et al., 2013). PPFD of 700 μmol·m^-2^·s^-1^ was the highest A_n_ obtained by Ke et al. (2022) compared to the intensity of 300 and 500 μmol·m^-2^·s^-1^. In our results, values of 700 and 1000 μmol·m^-2^·s^-1^ were the highest A_n_ obtained, too.

However, at similar intensity levels, the effect of the spectrum greatly influences A_n_ (**Table 5** and **Figure 3**). The best results were shown by the combination of red and blue LEDs (20B80R and 80R20R). Similar results were reported on tomato seedlings by (Hernández et al., 2016) after studying various spectra, concluding that the combinations of 30B70R and 50B50R showed a greater fresh and dry mass. However, there were no differences in A_n_ between the different spectra. Liu et al. (2011) indicated that the spectrum with the best performance in improving photosynthesis for tomato seedlings was the combination of RB in a 1:1 ratio with PPFD of 320 μmolm^2^s^-1^. Kaiser et al. (2019) indicated that in greenhouse tomato production, the optimal proportions of blue light are between 6-12%, while the higher values are the optimal plant growth. Liu et al. (2011) showed that of the monochromatic lights tested (blue, green, yellow and red), the one that showed the best behaviour was a blue light, coinciding with the results shown in this study (**Table 7**). Our results indicate that Blue light is more efficient in driving photosynthesis when comparing the three monochromatic light sources (100B>100R>100G). At the same time, photosynthesis is more efficient when Blue light is in combination with other colours, being the predominant wavelength of the mix. The absorbance values for Blue and Red light are between 80 and 95% (Terashima et al., 2009). Moreover, the limitation in one of these spectra causes photosynthesis inefficiency or other plant disorders (Hogewoning et al., 2010). This study has shown that monochromatic Red light impairs the photosynthetic machinery, reducing photosynthetic capacity in the so-called “red light syndrome” (Kaiser et al., 2019). This effect can be reverted by adding even small proportions of Blue light (Hogewoning et al., 2010). The peaks at which the LEDs used in this work emit light are closer to the absorption peak of chlorophylls in blue than in red, thus more effectively used by these pigments. This fact could explain the results obtained.

Greenlight has been proposed to drive photosynthesis more efficiently than Blue and Red light when light intensity reaches a saturating point (Terashima et al., 2009) due to the better distribution/penetration along the leaves. This effect is effectively used along the depths of the leaf and not only on the adaxial parts. However, this was not the case in this study. Greenlight reaches lower A_n_ values than Red and Blue light. Although, it is observed that at low intensities, the differences of A_n_ between Green and other spectra are more significant as the intensity of light increases (**Table 6**). This result could be because light saturating points have not been reached in this experiment, so all light received by leaves did not saturate the chloroplasts present on the adaxial part of leaves.

Further research should be performed at higher light intensities to determine whether higher intensities are needed to boost Green photosynthetic efficiency in tomatoes or whether this phenomenon is species-dependent and does not occur in tomato plants. One of the most critical variables in artificial lighting is PLUE, which represents the ratio between net photosynthesis and moles of photons applied. Concerning our test, it is observed that as the intensity increases, the PLUE

decreases. The values and trend shown align with those obtained by (Ke et al., 2022) with values between 30-40 with light intensities from 300 to 500 μmol·m^-2·^s^-1^ and CO_2_ concentrations of 1000 ppm.

The model established in this study does not adjust properly to the cases of lower light intensity (30 and 90 µmol·m-2·s-1), showing a discreet but lineal increase of An. This might be because the CO2 saturating point is reached at low light intensities, thus skipping the exponential part of the CO2 response curves. This would be in synchrony with the assumption of not reaching the light saturation point, evidencing a high light necessity of tomato (or at least the variety studied).

## 5 Conclusion

The interaction between light intensity and CO_2_ concentration on tomato seedlings has shown characteristic curves A_n_/Light and A_n_/CO_2_ for all spectra. The intensity of light and the concentration of CO_2_ are the parameters that most condition the A_n_ rate. The generated model and its parameters allow for the estimation and discrimination of the values achieved based on intensity, spectra, and CO2 concentration. For some fixed values of CO_2_ concentration and with close tested light intensities, spectra with better behaviour than others have been observed, and the differences between spectra with lower light intensities were more pronounced. The spectra with better behaviour, with a higher rate of A_n_, have been 20B80R and 80B20R. The tests carried out indicate that at low lighting intensities tested<350 μmol·m^-2^·s^-1^, the effect of the spectrum is more important because these increases represent a very high percentage with respect to the maximum potential of A_n_. In the artificial light application industry, where the intensities are low and can never compete with those coming from natural light, spectrum choice is essential to optimize the photosynthesis of the species, as indicated by the data on photosynthetic light use efficiency in this study. It is necessary to conduct more research to evaluate the growth and development of the complete plant since, although the spectra cited (20B80R, 80B20R) show better behaviour in A_n_, they can influence the morphology and growth of the plant in different ways from a crop perspective.

## Data availability statement

The raw data supporting the conclusions of this article will be made available by the authors, without undue reservation.

## Author contributions

RM: Conceptualization, Formal analysis, Funding acquisition, Investigation, Methodology, Supervision, Validation, Writing – original draft, Writing – review & editing, Resources, Visualization. RJ: Data curation, Formal analysis, Investigation, Writing – original draft. MMa: Data curation, Formal analysis, Project administration, Software, Visualization, Writing – original draft. MI: Data curation, Formal analysis, Methodology, Software, Validation, Writing – original draft. MMo: Conceptualization, Methodology, Resources, Writing – original draft. AT: Funding acquisition, Resources, Supervision, Validation, Visualization, Writing – review & editing, Writing – original draft.
